# Pentraxin 3 Plasma Levels and Disease Activity in Systemic Lupus Erythematosus

**DOI:** 10.1155/2015/354014

**Published:** 2015-11-03

**Authors:** Roberto Assandri, Marta Monari, Anna Colombo, Alessandra Dossi, Alessandro Montanelli

**Affiliations:** ^1^Clinical Investigation Laboratory, Humanitas Clinical and Research Center, Rozzano, Milan, Italy; ^2^Humanitas Clinical and Research Center, Rozzano, Milan, Italy; ^3^Clinical Investigation Laboratory, Diagnostics Department, Spedali Civili of Brescia, Brescia, Italy

## Abstract

SLE is an autoimmune disorder that involves polyclonal autoimmunity against multiple autoantigens. PTX3, a marker of the acute-phase inflammatory response, plays an important role in innate immunity and in modulation of the adaptive immune response. Our study tried to resolve some rather controversial aspects of the use of PTX3 as a biomarker of disease activity in SLE patients. We demonstrated that plasma PTX3 concentration of the SLE patients was significantly higher than the healthy control groups and reflected disease activity. ROC curve analysis was used to determine best cut-off point (2.8 ng/mL) with a good sensitivity and specificity. In patients with SLE, PTX3 concentrations were correlated with SLEDAI. Trend to remission (TTR) curve was created by plotting PTX3 levels and SLEDAI and we applied the curve as a model for the analysis of two patients with different follow-up. PTX3 plasma levels declined significantly and this decline occurred parallel to the clinical improvement with a complete remission of disease. In patients who experienced a clinical relapse, an increase in PTX3 levels followed the lupus flare. The proposal of PTX3 cut-off associated with TTR and monitoring of PTX3 plasma levels could be an innovative approach to follow-up of SLE patients.

## 1. Introduction

Systemic lupus erythematosus (SLE) is an autoimmune disorder with unpredictable course that involves polyclonal autoimmunity against multiple autoantigens and presents a wide spectrum of clinical manifestations (fever, skin rashes, arthralgia, and inflammation of kidney, lungs, or brain) [1]. The pathogenesis of SLE is based on combinations of genetic variants and environmental factors that promote loss-of-tolerance or tissue inflammation [2]. In fact there is growing evidence to suggest that inflammation and related molecules play a key role in the pathogenesis of SLE.

The pentraxin superfamily (PTXs), divided into long and short PTXs, can be induced by a variety of inflammation-associated stimuli [3]. Next to the classical short pentraxins C-reactive protein (CRP) and serum amyloid P component (SAP), pentraxin 3 (PTX3) is prototypic of long pentraxin family, a multifunctional protein characterized by a cyclic multimeric structure and a conserved domain [4].

An increasing number of studies have identified PTX3 as a key component in the host defence against certain infections such as fungal, bacterial, and viral ones [5, 6]. Furthermore, higher circulating PTX3 levels have been observed in cardiovascular diseases [7] and also in some autoimmune disorders, such as vasculitis [8], celiac disease (CD) [9], and SLE [3].

Despite several studies, the literature provides a contradictory description of PTX3 in SLE patients and, at the moment, it has not identified any biochemical markers that allow accurately monitoring disease activity.

Considering that SLE is characterized by chronic inflammation and immune dysfunction, PTX3 may play a role in the pathogenesis of this disease. Unlike CRP and SAP, PTX3 is produced by resident and innate immunity cells in peripheral tissues, in response to inflammatory signals [10].

However the literature provides a contradictory picture of PTX3 in SLE pathogenesis and several questions should be answered.

Our study tried to resolve some rather controversial aspects of the use of PTX as a biomarker of disease activity in SLE patients and finally proposed a model related to clinical use and follow-up.

## 2. Patients and Methods

### 2.1. Subjects

We studied 64 patients with SLE and 60 controls. We enrolled patients aged more than 18 years fulfilling the American College of Rheumatology (ACR) criteria for SLE [11]. Control subjects who did not meet the criteria for SLE or any other inflammatory disease and did not have infectious diseases were recruited.

Exclusion criteria of enrolled SLE patients were as follows: age > 50 years, pregnancy or postpartum patients, malignancy ischemic heart disease, concurrent infections, and recent trauma.

All patients subscribed written informed consent.

### 2.2. Clinical Assessment

Clinical information was obtained through a structured review of the medical records and laboratory tests. Subjects were considered to have hypertension if they took antihypertensive agents if they had a systolic blood pressure of at least 140 mmHg or a diastolic pressure of at least 90 mmHg.

The activity of SLE was measured with the SLE Disease Activity Index (SLEDAI) [11].

Antiphospholipid syndrome (APS) patients were enrolled following classification criteria established by international consensus document (2006): vascular thrombosis, one or more clinical episodes_ of arterial, venous, or small vessel thrombosis, Lupus Anticoagulant (LA) present in plasma, anticardiolipin (aCL) antibody of IgG and/or IgM isotype in serum or plasma, and anti-b2 glycoprotein-I antibody of IgG and/or IgM present in medium or high titer, on two or more occasions at least 12 weeks apart [12]. Patients with cerebrovascular accident, multi-infarct dementia, cerebral venous thrombosis, and transverse myelopathy were excluded.

Lupus nephritis was defined as present if the clinical and laboratory findings had met the ACR criteria (persistent proteinuria 0.5 mg/day or greater than 3+ by dipstick and/or cellular casts, including red blood cells, hemoglobin, granular, tubular, or mixed casts, and renal biopsy, according to the ISN/RPS 2003 classification [13]).

## 3. Methods

### 3.1. Blood Samples

Venous blood samples were collected after a minimum of 4 hours of fasting. The samples for PTX3 level analysis were stored at −75°C in small specimen containers.

### 3.2. Measurement of Plasma PTX3 Concentrations

The levels of PTX3 were measured by a sandwich ELISA kit (Hycult), on automated platform DSX (Technogenetics). Each sample was tested in duplicate with a dilution of 1 : 4 and the reported value refers to the mean of the two determinations. Both intra- and interassay coefficient of variation (CV) do not exceed 3%. The assay does not cross-react with CRP or serum amyloid A protein. Briefly, polystyrene microplates coated with a monoclonal antibody against PTX3 were incubated with serum samples for 1 hour at 37°C. For detection, biotinylated tracer polyclonal antibody specific to PTX3 was added for 1 hour and a streptavidin-peroxidase conjugate was added for 1 h. Color reaction was developed using tetramethylbenzidine and hydrogen peroxide. Optical density at 450 nm. was measured with a plate reader, and absolute values were calculated from the four-parameter logistic standard curve.

### 3.3. Statistical Analysis

Demographic characteristics were presented as the mean ± SD (median) for continuous variables and as frequencies and percentages for categorical variables. PTX3 concentrations were compared between SLE patients and healthy controls by using Wilcoxon's rank sum test.

The associations between the PTX3 level and SLEDAI or clinical characteristics CRP, ESR, anti-dsDNA antibody, C3, C4, and other laboratory parameters were examined in the SLE patients using Spearman's rank correlation analysis. Spearman's rank correlation coefficients were calculated to assess univariate associations between plasma PTX3 and continuous variables. All analyses in this study employed a significance level of *p* < 0.05 (two-sided). Cut-off value for PTX3 was selected from ROC curve analysis. Positive predictive value was calculated as (PPV = (true positive *∗* 100)/(true positive + false positive)) and negative predictive value was calculated as (NPV = (true negative *∗* 100)/(true negative + false negative)) and Youden's Index for the estimated cut-off point was calculated as sensitivity + specificity − 1.

Accuracy is measured by the area under the ROC curve.

## 4. Results

### 4.1. Characteristics of Patients and Healthy Control Subjects

Table 1 shows the baseline characteristics of the SLE patients and the control subjects.

In this study, 64 patients with SLE and 60 healthy controls were matched for age (mean ± SD 46.8 ± 15.1 years versus 41.8 ± 12.6 years, *p* = 0.801) and sex (percentage of women 87.5 versus 90%, *p* = 0.92).

In the patients' group, 15 patients had secondary form of APS and 15 patients had lupus nephritis (LN). There was no significant difference of either systolic blood pressure or diastolic blood pressure between the two groups (*p* = 0.78).

Patients with SLE had the same BMI (*p* = 0.023) as healthy control subjects and there were no significant differences with respect to smoking, history of coronary heart disease, and postmenopausal status between the two groups.

We investigated lipid profile of SLE patients and control subjects. The mean total cholesterol at diagnosis of celiac disease was 188 ± 75 mg/dL and HDL-cholesterol was 34 ± 15 mg/dL (Table 1).

Both men and women with SLE had the same HDL and triglycerides in comparison to healthy control (*p* = 0.54).

Patients were at different stages of disease activity with or without major organ involvement. The frequency of SLEDAI components in these patients was as follows: renal involvement: 23.4%, seizure: 6.4%, psychosis: 1.5%, severe headache: 5.4%, myositis: 5.4%, arthritis: 33.9%, malar rash/alopecia: 13.4%, APS: 18.4%, retinal vasculitis: 1.5%, anemia: 48.9%, lymphopenia: 40%, leukopenia: 11%, and thrombocytopenia: 14.9% (see Table 1).

The mean SLEDAI in these patients was 8 ± 3.5, ranged from SLEDAI = 0 to SLEDAI = 16.

In this study, patients were divided into three groups according to SLEDAI score: inactive group (1, 18 patients, 28%: SLEDAI < 4), active group (2, 32 patients, 50%: 4 < SLEDAI < 10), and highly active group (3, 14 patients, 22%: SLEDAI > 10).

### 4.2. Baseline Analysis

**Figure 1:**
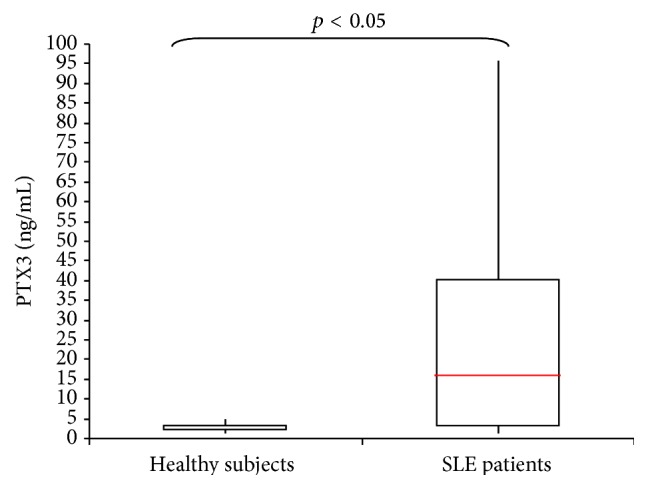
PTX3 plasma levels in SLE patients and healthy subjects.

Figure 1 shows the PTX3 plasma levels in SLE patients and control subjects.

In the SLE patients PTX3 plasma levels ranged from 1.18 to 95.1 ng/mL, with a mean ± SD of 14 ± 13.1 ng/mL and a median value of 5.3 ng/mL. In the control subjects, PTX3 ranged from 0.2 to 4.8 ng/mL, with a mean ± SD of 2.3 ± 1.1 ng/mL and a median value of 2.5 ng/mL. The plasma PTX3 concentration was significantly higher in the SLE patients than in the healthy controls (*p* < 0.001).

**Figure 2:**
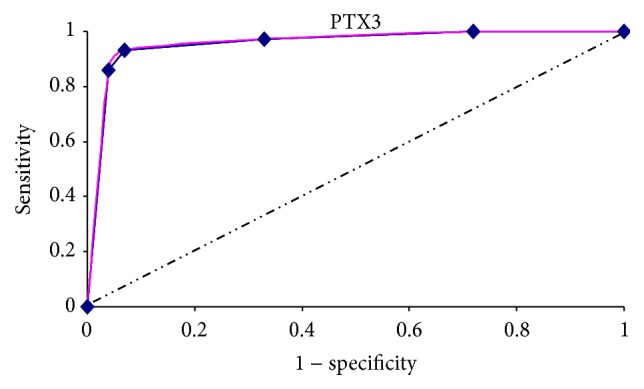
ROC curve analysis was used to determine PTX3 cut-off point between lupus patients and healthy controls. Example of a receiver operating characteristic curve. Solid red: ROC curve; dashed line: chance level; vertical line: (J) maximum value of Youden's Index for the ROC curve.

ROC curve analysis was conducted to determine a cut-off point for PTX3 plasma value between SLE patients and HS group. In the ROC curve shown PTX3 levels were plotted for their ability to predict the disease activity with true positive on vertical axis (sensitivity) and false positive (1 − specificity) on horizontal axis (Figure 2).

The estimated best cut-off point for PTX3 between active SLE patients and healthy subjects was 2.8 ng/mL, characterized by a high sensitivity (100%) and high specificity (80%), with a negative predictive value of 1 (100%), positive predictive value of 0.76 (76%), and an accuracy of 0.88 (88%).

Youden's Index was determined as 0.8. (see ROC curve in Figure 2).

## 5. Plasma PTX3 Concentration in SLE Patients

**Figure 3:**
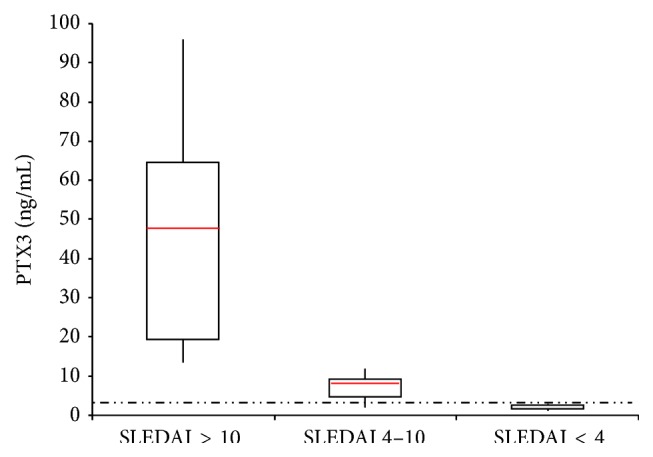
PTX3 plasma levels and SLEDAI patients categories. The dashed line represents cut-off value.

Figure 3 shows the distribution of patients according to PTX3 plasma values in groups A, B, and C.

PTX3 plasma concentration in group 1 ranged from 1.18 to 2.7 ng/mL with a mean range ± SD of 1.8 ± 0.60 ng/mL. PTX3 plasma concentration in group 2 ranged from 2.7 to 11.3 ng/mL with a mean range ± SD of 9 ± 2.4 ng/mL. Finally PTX3 plasma concentration in group 3 ranged from 13.5 to 95.9 ng/mL with a mean range ± SD of 47 ± 27.6 ng/mL (see Figure 2). There was a significant difference between the 3 groups (*p* < 0.001).

When patients were divided into two groups, according to calculated diagnostic cut-off point for plasma PTX3 (2.8 ng/mL), there was significant difference between active SLEDAI score and its individual parameters in these two groups (*p* = 0.001). Distribution of patients according to PTX3 cut-off point, in three groups of inactive, moderately active, and highly active patients, shows significant difference in PTX3 plasma levels in each group.

**Figure 4:**
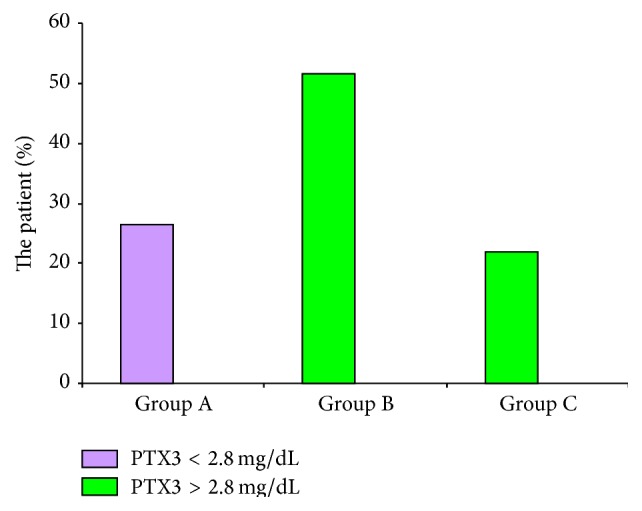
Distribution of patients according to PTX3 cut-off point in three groups of patients.

In patients with PTX3 < 2.8 ng/mL, 28% had SLEDAI < 4. In patients with PTX3 > 2.8 ng/mL, 72% had SLEDAI > 4 (see Figure 4).

## 6. Plasma PTX3 Concentration and Disease Activity

### 6.1. Complement System Fractions and CRP

The complement system was closely linked to SLE. All patients were tested to C3 and C4 fractions.

C3 and C4 serum concentration were estimated, respectively, in 825 ± 321 mg/L and 130 ± 60 mg/L.

The proportions of patients with low levels of complement fractions (C3 fraction < 900 mg/L, C4 fraction < 160 mg/L) were, respectively, 48 (75%) and 35 (54.6%).

The PTX3 plasma concentrations were compared in SLE patients with or without normal C3 and C4 complement fractions and in healthy subjects. There was a statistical difference in PTX3 plasma concentrations between the proportions of patients with low C3 and C4 fraction levels (16.5 ± 11.3 ng/mL), patients with normal levels (6.5 ± 2.7 ng/mL), and HCS. According to these data we examined correlation between plasma PTX3 concentration and complements system levels. There was a significantly negative correlation between C3 and C4 fractions and PTX3 plasma levels (resp., *r* = −0.34,  *p* = 0.02, and *r* = −0.42,  *p* = 0.002).

CRP serum levels were estimated in the three groups mentioned above (groups 1, 2, and 3).

CRP serum levels in SLE patients were estimated in 16.1 ± 15.3 mg/L, ranged from 0.1 to 83 mg/L with a median range of 9.1 mg/L. In group 1 CRP serum levels ranged from 0.1 to 9 mg/L with a mean value ± SD estimated in 6 ± 3.8 ng/L. In group 2 CRP serum concentration ranged from 0.2 to 14.3 mg/L (mean value ± SD 7.2 ± 5.2 mg/L) while group 3 ranged from 8.5 to 51 mg/L (mean value ± SD 28.1 ± 12.4 mg/L).

There are statistical differences between group 1 versus group 3 and group 2 versus group 3 and HC (*p* < 0.05), but no difference appeared between group 1 and group 2 (*p* = 0.62).

In light of these data we examined correlations between CRP serum concentration and PTX3 plasma levels. No correlation appeared between CRP and PTX3 levels (*r* = −0.08, *p* = 0.84).

### 6.2. Other Laboratory Biomarkers

We examined correlations between PTX3 plasma levels and other laboratory parameters in 64 patients with SLE. PTX3 plasma concentration was negatively correlated with serum albumin (*r* = −0.57, *p* = 0.01) and hemoglobin (*r* = −0.42, *p* < 0.05). Consequently PTX3 was still significantly associated with anemia (*p* = 0.020).

However, PTX3 was not correlated to serum creatinine, ESR, white blood cell count, lymphocyte count, and platelet count. Regarding immunological parameters no correlation appeared between PTX3 concentrations and anticardiolipin (aCL) immunoglobulin isotypes G (IgG), M (IgM) and IgG, IgM anti-b2 glycoprotein-I (anti-b2GPI) or anti-dsDNA antibody titer.

There was no correlation between the PTX3 concentration and several coronary risk factors, including serum cholesterol levels (total cholesterol, HDL-cholesterol, and LDL-cholesterol). Instead PTX3 was positively correlated with triglycerides (*r* = 0.39, *p* = 0.024; see Table 2).

## 7. Medication

The median dose of prednisolone was estimated in 1.8 (0–13) mg/day, although there was significant correlation between PTX3 plasma levels and prednisolone dosage (*r* = 0.49,  *p* < 0.05).

Twenty-two (36.6%) patients received cytotoxic drugs including cyclophosphamide (16.4%), azathioprine (13.5%), and mycophenolate mofetil (6.7%).

### 7.1. SLEDAI, “Trend to Remission Curve,” and Follow-Up of Patients

We considered a hypothetical correlation between disease activity, CRP serum concentration, and PTX3 plasma level. Activity patients categories have been defined on the base of SLEDAI scores as mentioned above.

We evaluated a correlation between SLEDAI and PTX3 plasma levels. We noted a positive significant correlation between SLEDAI and PTX3 concentration (*r* = 0.79, *p* < 0.001).

No significant correlation appeared between SLEDAI and CRP.

**Figure 5:**
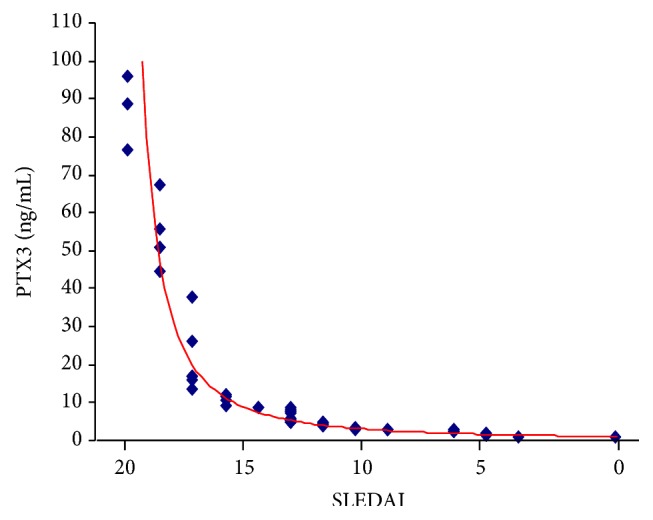
Trend to remission curve.

**Figure 6:**
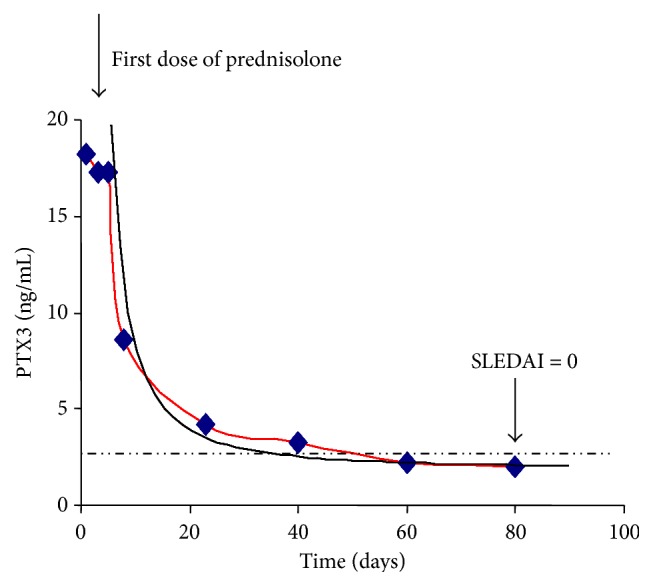
Patient 1 trend to remission. The dashed line represents the operative cut-off.

**Figure 7:**
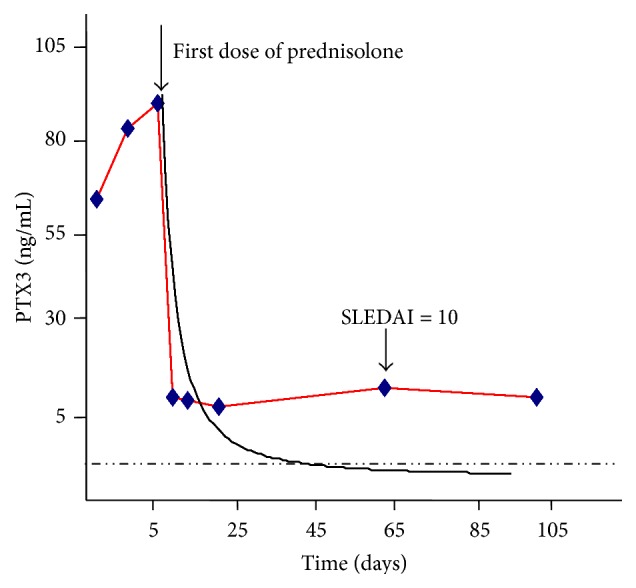
Patient 2 trend to remission curve. The dashed line represents the operative cut-off.

According to the evidences relationship between PTX3 plasma levels and SLEDAI we tried to provide a “trend to remission curve” (TTR curve, Figure 5). In the TTR curve PTX3 levels were plotted on vertical axis and SLEDAI on horizontal axis. In this way we linked the trend of SLE activity to PTX3 plasma concentrations. After that, we applied TTR curve as a model to the analysis of 2 patients, called Patient 1 (P1, Figure 6) and Patient 2 (P2, Figure 7), with two different follow-ups.

In P1, after initiation of high-dose corticosteroid therapy, PTX3 plasma levels declined significantly (from 18.2 to 2.1 ng/mL). This decline occurred parallel to the clinical improvement.

In this patient we showed a complete remission of disease (from SLEDAI 12 to SLEDAI 0, see Figure 6). We applied the TTR curve to the disease trend and we observed a complete match between TTR curve and the Patient's trend curve.

PTX3 plasma concentration of P1 was shown in Figure 6.

In P2 who experienced a clinical relapse, an increase in PTX3 plasma levels followed the relapse (see Figure 7). We applied the TTR curve to the patient's trend and we observed no complete match between the two curves.

## 8. Relationship between PTX3 and Organ Damage in SLE Patients

Since there was a positive correlation between the PTX3 plasma concentration and the disease activity indexes, we suspected that patients with major organ damage such as APS lupus or lupus nephritis could have higher PTX3 levels. Therefore, we compared PTX3 plasma concentrations between fifteen patients with LN and other APS-SLE patients. No statistical differences appeared between the two groups. We found that the PTX3 plasma concentration of the SLE patients with APS lupus (mean 22.2 ± 20.6 ng/mL) and LN was significantly higher than mean of LES patients (mean 24.2 ± 20.3 ng/mL).

In APS-SLE patients no correlation appeared between APS-specific autoantibodies and PTX3 plasma concentration. On the other hand plasma levels PTX3 was correlated with urine albumin and spot proteinuria in patients with LN (*r* = 0.62, *p* < 0.05; *r* = 0.67, *p* < 0.05).

## 9. Discussion

Pentraxins are a family of multimeric proteins divided into short and long pentraxin, based on the primary structure: CRP, the prototype of the short pentraxin subfamily, and PTX3, the prototypic long pentraxin.

Our study tried to resolve some rather controversial aspects of the PTX use as a biomarker of disease activity in SLE but despite several studies, the literature provides a contradictory picture of PTX3 in SLE patients and several questions should be answered.

It is now known that CRP levels, usually parallel with disease activity in inflammatory states, are almost never accompanied by elevated CRP levels [14]. Median CRP levels in patients with active RA are commonly in the range of 20–40 mg/L [14]. In contrast to this, Becker et al. and Borg et al. found median CRP levels during SLE exacerbation to be only 15 mg/L [14].

Literature provides evidences that hepatic CRP protein synthesis is inhibited by IFN*α*, which could explain the lack of correlation between inflammation and CRP in SLE. In support of this evidence, it is now known that CRP are associate with SLE disease activity in patients without measurable IFN*α* and without a genetic variant of the CRP gene associated with low levels of CRP [15]. Taken together, these findings have led to the conclusion that the CRP response in lupus is less than what is expected and CRP serum concentrations were not correlated with disease activity or with any types of clinical manifestations [14]. It is a reasonable research question to check if other pentraxins can overcome this limitation of CRP use as a biomarker of systemic disease activity in SLE.

Our study emphasized the concept that CRP serum levels did not correlate with disease activity and we have demonstrated that plasma PTX3 concentration correlated with disease activity and SLEDAI.

It has been demonstrated that Toll-Like Receptor (TLR) 7 (TLR7) and TLR9 signaling play a pivotal role in SLE pathogenesis. Very recent studies revealed that estrogen receptor *α* knockout mice have impaired inflammatory responses to TLR3, TLR4, TLR7, and TLR9 ligand stimulation in Dendritic Cells (DCs) [16, 17]. Also data in mice indicate that TLR4 plays a key role in mediating autoimmunity, proinflammatory cytokine production, and other immune activation.

TLR4 is one of the best characterized and the first member of the TLR family [18].

TLR4 signaling is implicated in the innate immune responses against a wide range of microbes, including Gram-negative and Gram-positive bacteria, mycobacteria, spirochetes, yeasts, and some viruses and mammary tumor viruses [17, 18].

TLR4 is implicated in a different range of pathological processes associated with autoimmune diseases such as psoriasis, diabetic retinopathy, thrombosis, and inflammatory disorders including arthritis and atherosclerosis [18].

There is an evident role of TLR4 in SLE pathogenesis, such as the kidney damage, the induction of CD40 and autoantibodies, the suppression of regulatory T cells, and the role of proinflammatory cytokines in SLE pathogenesis [19].

In mouse-model sex hormones could directly change TLR4 responsiveness through several mechanisms. Evidences suggested that NF*κ*B was a focal point in the signal transduction cascades that mediate inflammatory cues from antigen receptors on T cells and B cells, 80, and from TLRs on cells of the innate immune system [20]. In addition to hypomethylation-driven activation of NF*κ*B-sensitive genes, other oxidation-induced alterations in transcription factor programs are implicated in SLE [21].

The corresponding PTX3 human gene is located on chromosome 3 band q 25. The proximal promoter shares numerous transcription factor binding sequences (such as NF-kB) and [20, 21] it has been demonstrated that NF-kB binding site is essential for PTX3 gene transcriptional response. Also PTX3 is produced in response to a variety of inflammatory signals mediated by TLR agonists IL-1 and tumor necrosis factor alpha (TNF-alpha) [20–22].

It was elegantly demonstrated that PTX3 was strictly necessary for NF-kB activation in intestinal reperfusion injury model and underlined a fundamental role of PTX3 in mediating tissue inflammation under sterile conditions [22].

An integrate viewpoint suggested that PTX3 responsiveness lupus is possible and it could be speculated that PTX3 may play a role as a biomarker of disease activity.

In Hollan et al. study healthy subjects showed a serum PTX3 level as 1.21 ± 0.59 ng/mL [7].

On the other hand Yamasaki reported the mean plasma PTX3 concentration around 2.00 ng/mL for healthy Japanese people [23]. These different values could be referred to the use of two different matrices (plasma and serum) during the detection of healthy control PTX3 concentration.

In Shimada et al. study the mean PTX3 plasma concentration of 53 healthy controls was 2.2 ± 1.1 ng/mL [24], which was nearly identical to that of the healthy subjects in Yamasaki et al.'s study [23].

Fazzini et al. [8] and Hollan et al. [7] reported serum concentrations PTX3 (not plasma) of 28 SLE patients and three SLE patients, respectively. The mean PTX3 serum concentration of the 28 SLE patients reported by Hollan et al. was 0.38 ± 0.50 ng/mL, which was lower than that of 1.00 ± 0.47 ng/mL in their healthy controls [7]. However, 12 of their 28 SLE subjects did not have active disease with SLEDAI at zero (SLEDAI = 0).

Our study defined PTX3 plasma value in healthy subjects (2.3 ± 1.1 ng/mL) according to other studies, the concentration of PTX3 in SLE patients at different SLEDAI stage, and most importantly an operative cut-off (2.8 ng/mL), characterized by a high sensitivity (100%) and high specificity (80%). Furthermore PTX3 concentration reflects disease activity and “trend to remission curve” is a “friendly” instrument to apply in the clinical routine and follow-up of SLE patients.

## 10. Conclusions

A SLE biomarker would be a stable molecule that is easy to sample and cheap to measure and that discriminates disease activity and flares.

According to our data we could consider PTX3 as a “single and integrate biomarker” in SLE, a multifactorial pathology with wide spectrum of clinical manifestations.

It is a new and interesting approach. Future studies are needed, but the road is drawn.

## Figures and Tables

**Table 1:** Characteristics of SLE patients and healthy controls.

	SLE patients (*n* = 64)	Healthy controls (*n* = 60)	*p* value
Clinical features			
Age (years)	46.8 ± 15.1	41.8 ± 12.6	0.801
Sex (% of women)	87.5	90	0.84
Systolic blood pressure (mmHg)	120 ± 18	114 ± 12	0.63
Diastolic blood pressure (mmHg)	72 ± 13	70 ± 12	0.54
Clinical manifestations			
Renal involvement	23.4	—	NA
Seizure	6.2	—	NA
Severe headache	4.6	—	NA
Myositis	4.6	—	NA
Arthritis	34.3	—	NA
Malar rash/alopecia	12.5	—	NA
APS	18.4	—	NA
Retinal vasculitis	1.5	—	NA
Anemia	48.4	—	NA
Lymphopenia	40	—	NA
Leukopenia	11	—	NA
Thrombocytopenia	15.6	—	NA
Laboratory parameters			
Total cholesterol (mg/dL)	188 ± 75	193 ± 27	0.57
HDL-cholesterol (mg/dL)	48 ± 9	53 ± 4	0.54
LDL-cholesterol	95 ± 2	98 ± 3	0.91
Triglycerides (mg/dL)	49 ± 18	54 ± 11	0.54
Serum iron (*μ*g/mL)	91 ± 14	98 ± 12	<0.05
Hemoglobin (g/dL)	8 ± 2	9.4 ± 4	<0.05
C3 (mg/L)	82.5 ± 32.1	102.5 ± 52	<0.05
C4 (mg/L)	13.1 ± 6.7	31 ± 12.1	<0.05
Anti-dsDNA (IU/mL)	814 ± 461	1 ± 0.2	<0.05
Serum creatinine (mg/dL)	1.2 ± 0.9	0.97 ± 0.2	<0.05
Serum albumin (g/dL)	2.8 ± 0.7	4.1 ± 0.4	<0.05
Inflammatory marker			
Erythrocyte sedimentation rate (mm/h)	58 ± 40	10 ± 12	<0.05

**Table 2:** Factors associated with plasma PTX3 levels in SLE patients.

Variables	Correlation coefficient	*p* value
Clinical features		
Age (years)	0.047	0.80
Systolic blood pressure (mmHg)	0.01	0.75
Diastolic blood pressure (mmHg)	0.10	0.64
BMI (Kg/m^2^)	0.11	0.41
Clinical manifestations		
Renal involvement	0.61	<0.05
Seizure	0.18	0.71
Severe headache	0.12	0.27
Myositis	−0.18	0.09
Arthritis	0.21	0.27
Malar rash/alopecia	−0.09	<0.05
APS	0.49	<0.05
Retinal vasculitis	0.03	0.59
Anemia	−0.42	<0.05
Lymphopenia	−0.13	0.43
Leukopenia	0.17	0.51
Thrombocytopenia	−0.17	0.41
Laboratory parameters		
Total cholesterol (mg/dL)	0.02	0.61
HDL-cholesterol (mg/dL)	0.12	0.51
LDL-cholesterol	0.12	0.42
Triglycerides (mg/dL)	0.03	<0.05
Serum iron (*μ*g/mL)	−0.41	<0.05
Hemoglobin (g/dL)	0.42	<0.05
C3 (mg/L)	−0.34	0.02
C4 (mg/L)	−0.42	0.002
Anti-dsDNA (IU/mL)	0.14	0.53
Serum creatinine (mg/dL)	0.02	0.80
Serum albumin (mg/dL)	−0.57	<0.05
AlbU (mg/dL)	0.61	<0.06
Inflammatory marker		
Erythrocyte sedimentation rate (mm/h)	0.23	0.09
C-reactive protein (mg/dL)	−0.08	0.84
Fibrinogen (mg/dL)	−0.11	0.41
